# An Optimal Parameter Selection Method for MOMEDA Based on *EHNR* and Its Spectral Entropy

**DOI:** 10.3390/s21020533

**Published:** 2021-01-13

**Authors:** Zhuorui Li, Jun Ma, Xiaodong Wang, Xiang Li

**Affiliations:** 1 Fauclty of Information Engineering and Automation, Kunming University of Science and Technology, Kunming 650500, China; kmustmcollzr@163.com (Z.L.); wangxd@kmust.edu.cn (X.W.); martinlx@163.com (X.L.); 2 Yunnan Key Laboratory of Artificial Intelligence, Kunming University of Science and Technology, Kunming 650500, China

**Keywords:** multipoint optimal minimum entropy deconvolution adjusted, envelope harmonic-to-noise ratio, *EHNR* spectral entropy, improved grid search, fault feature extraction

## Abstract

As a vital component widely used in the industrial production field, rolling bearings work under complicated working conditions and are prone to failure, which will affect the normal operation of the whole mechanical system. Therefore, it is essential to conduct a health assessment of the rolling bearing. In recent years, Multipoint Optimal Minimum Entropy Deconvolution Adjusted (MOMEDA) is applied to the fault feature extraction for rolling bearings. However, the algorithm still has the following problems: (1) The selection of fault period *T* depends on prior knowledge. (2) The accuracy of signal denoising is affected by filter length *L*. To solve the limitations, an improved MOMEDA (IMOMEDA) method is proposed in this paper. Firstly, the envelope harmonic-to-noise ratio (*EHNR*) spectrum is adopted to estimate the fault period of MOMEDA. Then, the improved grid search method with *EHNR* spectral entropy as the objective function is constructed to calculate the optimal filter length used in the MOMEDA. Finally, a feature extraction method based on the improved MOMEDA (IMOMEDA) and Teager-Kaiser energy operator (TKEO) is applied in the field of rolling bearing fault diagnosis. The effectiveness and generalization performance of the proposed method is verified through comparison experiment with three data sets.

## 1. Introduction

Rolling bearings are vital components of mechanical systems and have been widely used in rotating machinery. However, rolling bearings often work in more demanding environments for a long time, such as corrosive, high temperature, large impact and high load conditions, which increases the uncertainty of the mechanical system, resulting in various fault defects of the bearings [1]. In order to avoid the environmental pollution, economic loss and even safety problems caused by rolling bearing shutdown, the fault diagnosis method based on signal analysis is widely used in industrial production, in which the bearing condition is recognized according to the effective information collected by the vibration signal [2,3], pressure signal [4], temperature signal [5] and acoustic signal [6,7].

The fault diagnosis technique based on vibration signal has been widely used in practice because of its intuitive operation and reliable diagnosis results, where the vibration signal of the bearing is monitored by the acceleration sensors installed in the appropriate direction of the bearing seat or the box, and the signal is analyzed and processed to determine the bearing condition. Furthermore, some advanced measurement systems are applied in rolling bearings. Wang [8] designed a data acquisition system of high speed railway bearing based on ZigBee technology. Gao et al. [9] designed a wireless sensor vibrating signal acquisition and processing system, and the researchers could collect and analyze the signal timely and conveniently. Li et al. [10] proposed a smart metasurface shaft (SMST) to achieve single-sensor identification of vibration sources, which showed potential applications in rotating machinery condition monitoring. Verstraete et al. [11] presented multi-sensor fusion systems for integrated remaining useful life prognostic capabilities. Meng et al. [12] developed a fully enclosed bearing-structured self-powered rotation sensor (SPRS) for multi-tasking motion measurement. Furthermore, the device displayed high precision, broad range, quick response, and excellent durability.

Fault feature extraction based on signal analysis is critical after the reliable vibration data source is available. In industrial production, when the bearing fails to work, the collected vibration signal will generate periodic transient pulses at a certain characteristic frequency [13]. However, in fact, in addition to the pulses caused by fault defects, there are also some modulation harmonics caused by external excitations, such as axial unbalance, misalignment, critical speed, structural resonance and uncertain factors on vibration signal transmission paths, which make the spectrum more complex and increases the difficulty of bearing fault diagnosis [14]. Therefore, recovering and enhancing effective fault period impulse components from vibration signals is of great significance and importance for fault feature extraction. Some signal processing methods have been successfully employed in capturing fault-related transient components in the non-stationary signal for rolling bearings [15], such as spectral kurtosis [16,17], wavelet transform theory [18] and so on. Unfortunately, most of the methods mainly concentrate on emphasize random impulse with large amplitude and thus ignores the periodicity of fault signal.

To tackle the limitation, the deconvolution principle has been introduced for bearing fault periodic composition extraction, which achieved satisfactory results. In this line, Wiggins [19] proposed the minimum entropy deconvolution (MED) in 1978, which took kurtosis as the objective function and the optimal inverse filter was iteratively found to enhance the transient ingredients in the original signals. In 2007, Randall successfully applied the MED to fault detection [20]. Since then, the MED has been widely used in the fault feature extraction of rotating machinery [21,22,23].

However, the MED method aims at maximizing the kurtosis value, and thus it is easily affected by strong noise. In the iterative process, only the pulse component in the vibration signal can be enhanced and this defect limits the development of MED method [24]. To address this issue, McDonald et al. [25] proposed the maximum correlation kurtosis deconvolution (MCKD), which took the correlation kurtosis as the objective function and iteratively searched for the optimal reverse filter to enhance the periodic transient components in the original signal. The MCKD algorithm has achieved remarkable results in the fault feature extraction of rotating machinery such as rolling bearings [26,27], gearboxes [25], and motor rotors [28]. Nonetheless, there are still some issues in the practical application of MCKD. In fact, its accuracy is affected by four parameters: filter length, fault period, the number of displacement and the number of iterations [29]. Moreover, the MCKD can only address the single period pulse signal.

To extract the continuous periodic pulse signal, McDonald et al. [30] proposed the multipoint optimal minimum entropy deconvolution adjusted (MOMEDA) method. This method is a non-iterative deconvolution process and utilizes infinite pulse sequence as the optimization object function to search for the optimal filter directly. With these advantages, the MOMEDA has been successfully applied to the rotating machinery fault characteristics extraction [31,32]. Ma et al. [33] introduced MOMEDA to detect planet bearings faults and the experiments show that MOMEDA presented specific applicability compared with the traditional kurtogram filtering method. Li et al. [34] suggested a method based on the MOMEDA and long and short time memory (LSTM) for fault diagnosis. Zhu et al. [35] introduced a method based on the MOMEDA and TKEO for the rolling bearing fault diagnosis with good experimental qualities. However, the following weaknesses of the MOMEDA method are also well noted in the community: (1) the performance of the MOMEDA mainly depends on the prior knowledge of fault period *T*. It is necessary to identify the periodic impulses signal through the multipoint kurtosis spectrum [30] in the default fault period *T* search interval. However, when the original signal is affected by complex background noise, the multipoint kurtosis spectrum may not provide correct fault period information according to the location of maximum multipoint kurtosis. (2) The effect of noise reduction for MOMEDA is also affected by the filter length *L*. Hence, unreasonable filter length *L* will weaken or enhance the energy of the original signal, and then cause misdiagnosis. To address the above-mentioned issues, scholars have introduced some optimization algorithms [36,37] to determine the fault period *T* and filter length *L* of MOMEDA, such as particle swarm optimization (PSO) algorithm [27,38], grasshopper optimization algorithm (GOA) [39] and so on. These parameter optimization algorithms mostly take kurtosis, envelope spectrum kurtosis (ESK) and other similar indexes as objective functions to improve MOMEDA. However, the methods also have some limitations. These algorithms are usually applied to multi-objective optimization, i.e., training parameters such as fault period *T*, filter length *L* and window function simultaneously, which make the implementation process complex and time-consuming. Therefore, it is necessary to explore other effective methods to quickly calculate the fault period *T* and filter length *L* of MOMEDA for improving the deconvolution performance.

An improved MOMEDA (IMOMEDA) method is investigated for fault feature extraction in this paper. The proposed method incorporates the envelope harmonic-to-noise ratio (*EHNR*) [40] into the MOMEDA scheme. By calculating the *EHNR* spectrum of the original signal, the fault period *T* can be quickly determined without using the prior information. Furthermore, kurtosis spectral entropy is proposed as a grid search objective function to search for the optimal filter length *L* of MOMEDA, for which the single objective optimization can greatly simplify the procedures [24]. However, kurtosis spectral entropy tends to emphasize random impulse with large amplitude (it inherits the essential characteristic of kurtosis) and thus ignores the periodicity of fault signals. Therefore, *EHNR* spectral entropy is presented as the target function to overcome this shortcoming and obtain the improved grid search method to select the optimal filter length *L* of MOMEDA. Compared with the existing methods, the overall approach has the following advantages:

(1) A new index-based *EHNR* spectrum is proposed to calculate fault period *T* of MOMEDA and require less prior knowledge and calculation time.

(2) *EHNR* spectral entropy is introduced to construct the objective function of grid search method and select the optimal filter length *L* of MOMEDA.

(3) A novel feature extraction method-based IMOMEDA-TKEO is presented and applied in the field of rolling bearing fault diagnosis.

(4) The effectiveness and generalization performance of the presented method is verified through comparison experiment with three data sets.

The remaining part of the papers is organized as follows: Section 2 introduces the basic principles of MOMEDA and *EHNR*. Section 3 elaborates on the implementation process of IMOMEDA-TKEO. Section 4 presents the experimental results and analysis. Section 5 gives the discussion and summary.

## 2. Theoretical Background

### 2.1. MOMEDA Method

Suppose *x* is the vibration signal, *y* is the impulse sequence, *h* is the transfer function, *e* is noise, and “*” is the convolution product. The discrete-time response signal is as follows [41]:

(1) x=h*y+e

The MOMEDA algorithm aims at finding an optimal filter *f* in a non-iterative way to realize the reconstruction of the original impulse sequence *y* and try to reduce the impact of noise. The major process of MOMEDA algorithm is given as follows:

(2) $$y=f*x=\sum_{k=1}^{N-L}f_{k}x_{k+L-1}$$

Or in matrix form:

(3) $${y=X}_{0}^{T}f$$

(4) $$X_{0}^{}=\left[\begin{array}{ccccc} x_{1} & x_{2} & x_{3} & ... & x_{N}\\ 0 & x_{1} & x_{2} & ... & x_{N-1}\\ 0 & 0 & x_{1} & ... & x_{N-2}\\ ... & ... & ... & ... & ...\\ 0 & 0 & 0 & ... & x_{N-L+1} \end{array}\right]$$

Among them, *k* = 1, 2,…, *N*−*L*, according to the periodicity of the fault signal for rotating machinery, MOMEDA takes the multi D-norm of the filtered signal as the objective function, the concept of MOMEDA is presented in Equations (5) and (6):

(5) $$\mathrm{MDN}(y,t)=\frac{1}{\left||t|\right|}\frac{t^{T}y}{\left||y|\right|}$$

(6) $$\mathrm{MOMEDA}=\underset{f}{max}\mathrm{MDN}(y,t)=\underset{f}{max}\frac{t^{T}y}{\left||y|\right|}$$

The target vector *t* is a constant vector that defines the location and weightings of the goal impulses to be deconvolved, which is suitable for the features extraction [19]. When *t* is completely consistent with original impulse signal *y*, the multi D-norm reaches the maximum value and the deconvolution effect works the best. Therefore, the non-iterative process of Equation (6) is formulated as follows:

(7) $$\frac{d}{df}\left(\frac{t^{T}y}{\left||y|\right|}\right)=0$$

(8) $$\frac{d}{df}\left(\frac{t^{T}y}{\left||y|\right|}\right)={\left\|y\right\|}^{-1}\left(t_{1}M_{1}{+t}_{2}M_{2}+...{+t}_{k}M_{k}\right)-{\left\|y\right\|}^{-3}t^{T}{yX}_{0}y=0$$

where *f* = *f*_1_, *f*_2_, *f*_3_,…*f_L_*, *t* = *t*_1_, *t*_2_, *t*_3_,… *t_N−L_*. *M_k_* is the *k*th sequence pulse to be deconvolution, it can be expressed as ${\overset{}{M}}_{k}=\left[\begin{array}{c} x_{k+L-1}\\ x_{k+L-2}\\ \cdot \cdot \cdot \\ x_{k} \end{array}\right]$, Suppose *X*_0_ = [*M*_1_,*M*_2_,…*M_K_*], the Equation (8) can be simplified as:

(9) $${\left\|y\right\|}^{-1}X_{0}t-{\left\|y\right\|}^{-3}t^{T}{yX}_{0}y=0$$

(10) $$\frac{t\cdot y}{{\left\|y\right\|}^{2}}X_{0}{y=X}_{0}t$$

We then substitute ${y=X}_{0}{}^{T}f$ into Equation (10), and then calculate:

(11) $$\frac{t\cdot y}{{\left\|y\right\|}^{2}}{f=(X}_{0}X_{0}{}^{T}{)}^{-1}X_{0}t$$

The ${f=(X}_{0}X_{0}{}^{T}{)}^{-1}X_{0}t$ can be denoted as a set of optimal filters, and then reconstruct the original impulse signal *y*:

(12) $$Y=[\begin{array}{cccc} y_{1} & y_{2} & \cdot \cdot \cdot & y_{M} \end{array}{]y=X}_{0}{}^{T}f$$

Finally, the multipoint kurtosis (MKurt) is defined as follows:

(13) $$\mathrm{MKurt}=(\sum_{n=1}^{N-L}t_{n}{}^{2}{)}^{2}\sum_{n=1}^{N-L}{{(t}_{n}y_{n})}^{4}/(\sum_{n=1}^{N-L}t_{n}{}^{8}(\sum_{n=1}^{N-L}y_{n}^{2}{)}^{2})$$

However, the construction of target vector *t* needs to take *T* as a prior knowledge. In practical engineering, it is usually impossible to meet this condition due to the complex working environments, which in turn limits the application of the MOMEDA method. In addition, the periodic pulse energy is determined by the filter length *L*. If the parameter setting is unreasonable, it will weaken or enhance the energy of the periodic impulse signal which will lead to misdiagnosis. Therefore, how to calculate the fault period *T* and filter length *L* for the MOMEDA method quickly is one of the major issues, aiming at improving its performance.

### 2.2. *EHNR* Principle

In the field of condition-based maintenance, a large number of indicators are introduced to determine whether the measured signal is informative or not. These indicators are widely used, including kurtosis, entropy to *L*2/*L*1 norm, Gini index and other norm indexes [42]. Most of the indexes mainly concentrate on the peak value, non­­-gaussianity distribution and other general statistical distribution, thus ignoring the particularity of mechanical signals. Therefore, this paper introduces the *EHNR* method. According to the specific characteristics of rolling bearing fault signal, we can determine the parameter *T* of MOMEDA quickly.

The calculation process of *EHNR* is as follows [40]:

(1) The envelope signal *Env_x_*(*t*) of vibration signal *x*(*t*) is obtained by Hilbert transform and the DC component is removed.

(14) $$\hat{x}(t)=H\{x(t)\}=\frac{1}{\pi }\int_{-\infty }^{\infty }\frac{x(\tau )}{t-\tau }d\tau$$

(15) $${Env}_{x}^{\prime }(t)=\sqrt{x^{2}(t)+{\hat{x}}^{2}\left(t\right)}$$

(16) $${Env}_{x}{(t)=Env}_{x}^{\prime }{(t)-\mathrm{mean}(Env}_{x}^{\prime }\left(t)\right)$$

(2) The autocorrelation function of *Env_x_*(*t*) is calculated as:

(17) $$r_{{Env}_{x}}(\tau )=\int {Env}_{x}\left(t\right){Env}_{x}(t+\tau )\mathrm{dt}$$

**Figure 1:**
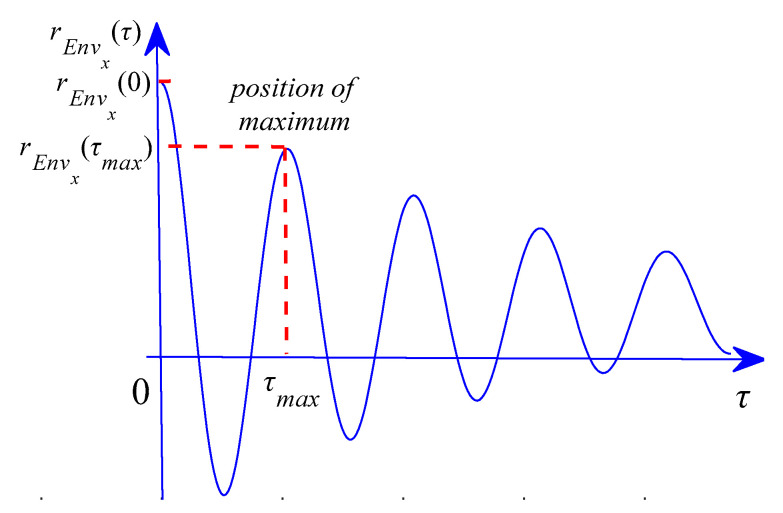
Autocorrelation function spectrum.

(3) The maximum position of the autocorrelation function of the signal is found in the lag domain, where the point is shown in Figure 1.

Then, the *EHNR* can be defined as Equation (18):

(18) $$EHNR=\frac{r_{{Env}_{x}}{(\tau }_{max})}{r_{{Env}_{x}}{(0)-r}_{{Env}_{x}}{(\tau }_{max})}$$

where *τ_max_* is the time lag that makes the autocorrelation spectrum of *Env_x_*(*t*) reach its local maximum, $r_{{Env}_{x}}{(\tau }_{max})$ is the energy of harmonic, $r_{{Env}_{x}}\left(0\right)$ is the total energy of envelope signal.

### 2.3. Setting Filter Length L Based on Grid Search Method

The grid search [43] is an exhaustive search method for specifying the accurate parameter values. According to the definite step size, the grids are divided in a certain range and the constraint function values on each grid point are calculated. Then, the independent variables that make the objective function value optimal (maximum or minimum) are selected as the solution of the problem. 

In this paper, the *EHNR* spectral entropy is proposed as the objective function and the expression is shown in Equation (19):

(19) $$EHNR\_E\left(x\right)=\frac{EHNR(x)}{E_{s}}$$

Among them, $\sum_{i=1}^{N}P_{i}{(S}_{R}\left(\omega )\right)=1$ and

(20) $$E_{s}=-\sum_{i=1}^{N}P_{i}{(S}_{R}\left(\omega )\right){\ln P}_{i}{(S}_{R}\left(\omega )\right)$$

where $S_{R}(\omega )$ is the envelope spectrum of the original signal, *N* is the data length, *EHNR*(*x*) represents the *EHNR* value of the input signal, and *EHNR*_*E*(*x*) represents the *EHNR* spectral entropy of the signal. For the periodic pulse signal, the envelope spectrum is concentrated on the low-frequency region which results in a smaller *E_s_* value. *EHNR*(*x*) is a function to describe the periodic change of faults. The larger the *EHNR*(*x*) is, the stronger the periodicity. Therefore, *EHNR*_*E*(*x*) can reflect the uniformity of the signal period.

In this paper, *EHNR*_*E*(*x*) is used as the objective function of grid search method, and then the optimal filter length *L* is determined.

### 2.4. TKEO Algorithm

The Teager-Kaiser energy operator is a nonlinear operator that can track the instantaneous energy of signal. The extraction of envelope by TKEO is to calculate the three adjacent sampling points of the measured waveform. TKEO can track the change of the measured signal waveform in real time. The algorithm has the advantages of excellent time resolution, simple and fast calculation. The specific mathematical expression and implementation process of the TKEO algorithm was proposed in [44].

## 3. The Proposed Method

**Figure 2:**
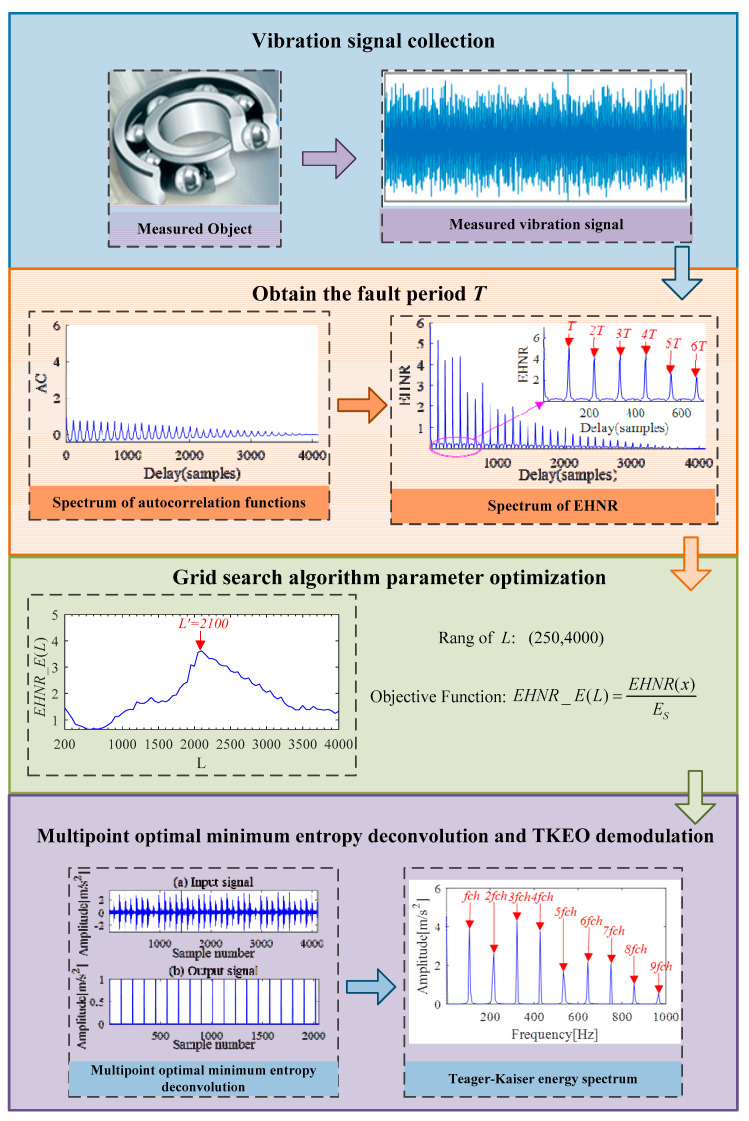
Implementation flow chart of IMOMEDA-TKEO method.

Inspired by the above-introduced methods, in this section, an IMOMEDA-TKEO rolling bearing fault feature extraction method is proposed. The framework of the proposed method is shown in Figure 2. The general implementation procedure is summarized as follows:

Step 1: The autocorrelation function $r_{{Env}_{x}}(\tau )=\int {Env}_{x}\left(t\right){Env}_{x}(t+\tau )\mathrm{dt}$ of rolling bearing vibration signal *x*(*t*) is obtained by the autocorrelation analysis.

Step 2: The envelope harmonic-to-noise spectrum *EHNR*(*x*) of signal *x*(*t*) is obtained and the fault period *T* is calculated.

Step 3: According to [45], the filter length *L* meets the principle of *L* > 2*f_s_* /*f_c_*, where *f_s_* is the sampling frequency and *f_c_* is the resonance frequency. We initialize the input parameters of the grid search, set the search range of the filter length *L* to (250, 4000), the search step length as 50, and then the fault period *T* is obtained by Step 2.

Step 4: The parameters *L* are updated by *EHNR* spectral entropy *EHNR*_*E*(*L*). When *EHNR*_*E*(*L*) reaches maximum, the optimal filter length *L* is obtained.

Step 5: The parameters of MOMEDA algorithm are set according to step 2 and step 4, and then the original signal *x*(*t*) is deconvolved to obtain the analysis signal *x_cov_*(*t*).

Step 6: The signal *x_cov_*(*t*) is demodulated by TKEO to get its Teager-Kaiser energy spectrum, and then complete fault feature extraction.

## 4. Experimental and Comparative Analysis

In order to verify the effectiveness of the IMOMEDA-TKEO method, the experimental database of rolling bearing from Case Western Reserve University (CWRU) [46], National Aeronautics and Space Administration (NASA) [47] which are authoritative in the field of bearing fault diagnosis and self-made experiment platforms (or KUST-SY for short) are used for experimental analysis. Comparative experiments among IMOMEDA-TKEO, MOMEDA-TKEO and fast kurtogram (FK) [48] are carried out to show the superiority of the proposed method. In each case, we utilize the same input data to complete each comparative experiment. The brief overview of the comparative experiment is shown in Table 1. In each case, we utilize the same input data to complete each comparative experiment.

(a) Compared with the other experiments, the Test 1 demonstrates the effectiveness of the IMOMEDA-TKEO method.

(b) The comparison between Test 2 and Test 3 proves that it is necessary and effective to optimize the filter length of MOMEDA.

(c) Compared with fast kurtogram, the other tests verify that the MOMEDA method can enhance effective fault period impulse component from vibration signals.

(d) The demonstration of pseudo period in Test 3 further proves the importance to calculate fault period *T* exactly.

### 4.1. Case 1: CWRU Data Analysis

**Figure 3:**
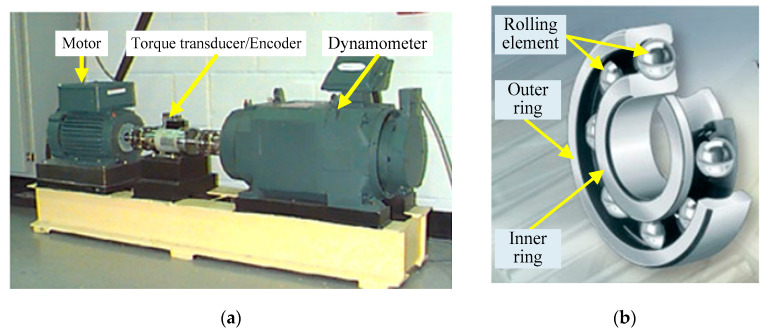
Bearing test rig of CWRU data center (**a**) The simulation experiment platform (**b**) The rolling bearing.

Figure 3 shows the experiment platform and bearings. Some specific measured parameters are presented in Table 2. The test-bed consists of a two horsepower motor, a torque sensor/decoder, a power tester and an electronic controller. The vibration signals were collected by accelerometers, where the accelerometers are installed at 12 o’clock position at drive end of the motor and fan end of motor housing, respectively. The collected signals were stored in a 16-channel DAT recorder [46] and post-processed in MATLAB 2018a on an IntelI CITM) i5-7300HQ CPU @ 2.50 GHz Laptop. The motor speed is 1797rpm. The sampling frequency *f_s_* is 12 kHz, and the number of data points *N* is 4096.

Outer race defect frequency:

(21) $$\mathrm{BPFO}=\frac{Z}{2}\left(1-\frac{d}{D}\cos\theta \right){\times f}_{r}$$

Inner race defect frequency:

(22) $$\mathrm{BPFI}=\frac{Z}{2}\left(1+\frac{d}{D}\cos\theta \right){\times f}_{r}$$

According to the above parameters and formulas, BPFO = 107.36 Hz and BPFI = 162.19 Hz are calculated, respectively. Next, we use the datasets in CWRU to complete the comparative experiments.

#### 4.1.1. Outer Race Fault Feature Extraction

**Figure 4:**
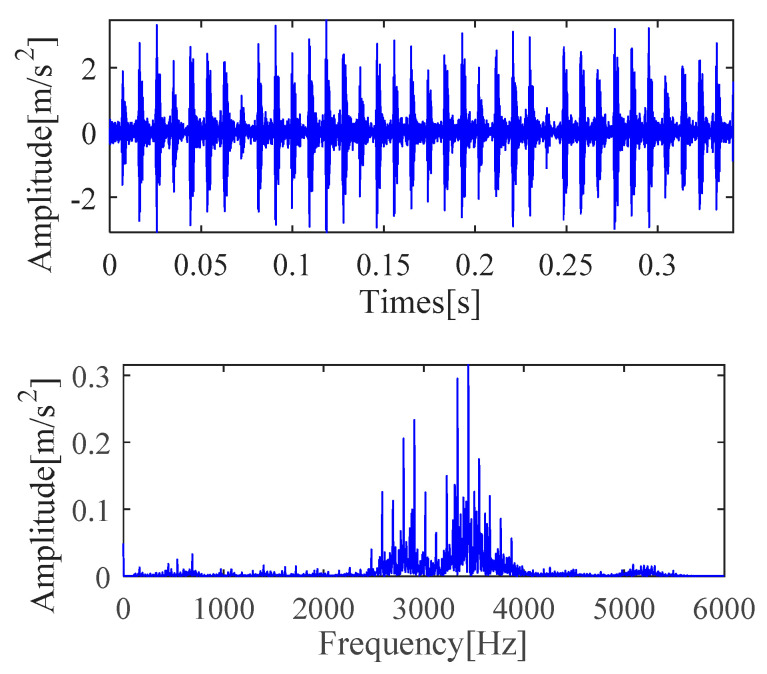
Vibration signal time and frequency analysis.

Figure 4 shows the time-domain and frequency-domain analysis results of the original outer race signal, respectively. It is seen from Figure 4 that these waveforms cannot directly obtain the information related to the outer race fault. Further analysis is required to determine the fault characteristic frequency and other details. Therefore, IMOMEDA-TKEO, MOMEDA-TKEO and the FK methods are proposed to complete subsequent analysis.

(A) IMOMEDA-TKEO method;

(a) Using the *EHNR* spectrum to calculate fault period *T.*

**Figure 5:**
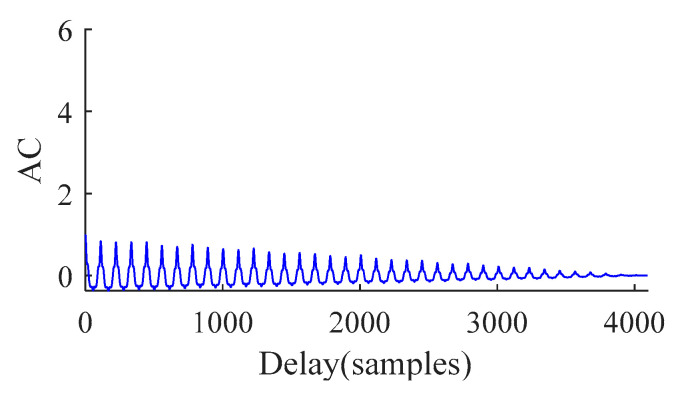
Autocorrelation function spectrum.

**Figure 6:**
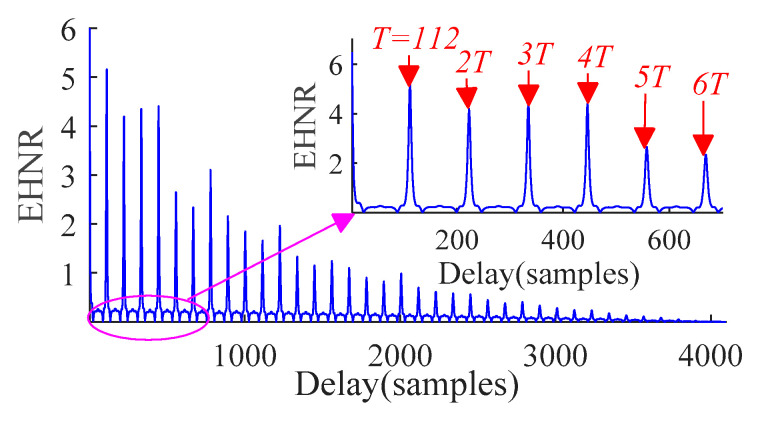
*EHNR* spectrum.

By carrying out the autocorrelation analysis of outer ring, the autocorrelation function (AC) spectrum of the original signal is obtained as shown in Figure 5. It can be seen from Figure 5 that the AC spectrum can reflect the periodic change of fault signal, which provides the basis for the estimation of fault period *T*. In order to estimate *T*, we further calculate the *EHNR* spectrum of signal as shown in Figure 6. It can be seen from Figure 6 that the fault signal shows obvious periodic change and gradually decays with time, which meets the trend of fault signal. Then, the fault period *T* = 112 is obtained.

(b) Using the *EHNR* spectral entropy to get filter length *L*

**Figure 7:**
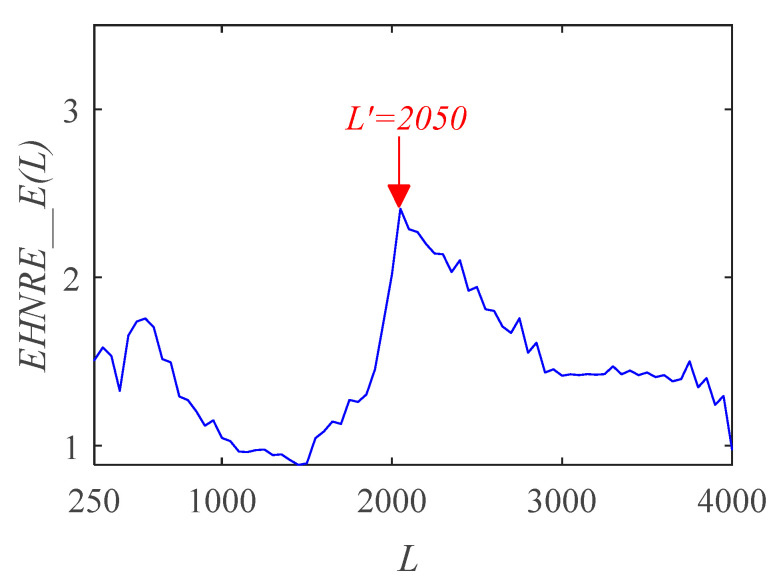
Trend chart of *EHNR* spectral entropy.

Given the fault period *T* = 112, the filter length *L* of the MOMEDA is quickly determined by the grid search method. Firstly, the filter length *L* satisfies *L* > 2*f_s_* /*f_c_*, where *f_s_* is the sampling frequency and *f_c_* is the resonance frequency [45]. To accurately cover the whole fault frequency band, the initialization *L* search range is (250, 4000) and the search step is 50. Secondly, the *EHNR* spectral entropy is proposed as the target function. Finally, the trend of *EHNR* spectral entropy is obtained by constantly updating the parameter *L* as shown in Figure 7. When obtaining the maximum output of *EHNR*_*E*(*L*), the optimal filter length is 2050.

(c) Outer race fault feature extraction

**Figure 8:**
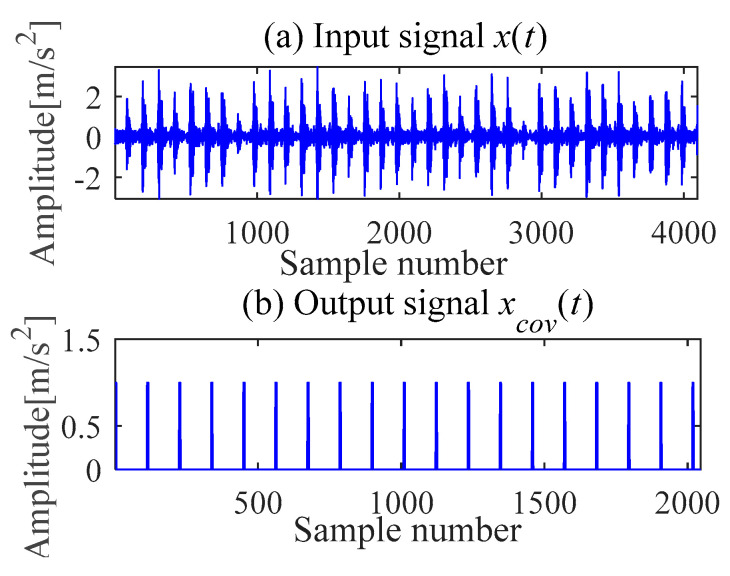
Signal deconvolution (IMOMEDA).

**Figure 9:**
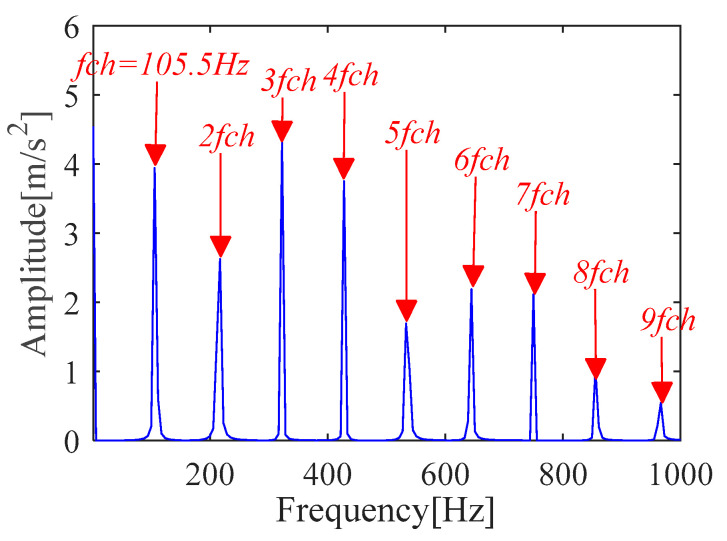
Teager-Kaiser energy spectrum (*x*_*_cov_*(*t*)).

According to (a) and (b), the fault period *T* and filter length *L* of MOMEDA are calculated as 112 and 2050, respectively. Then, the periodic impulse signal *x_cov_*(*t*) of the original signal *x*(*t*) is extracted by using the MOMEDA method, as shown in Figure 8. Then, we demodulate *x_cov_*(*t*) with TKEO and get the corresponding spectrum as shown in Figure 9. It can be seen from Figure 9 that the characteristic frequency (105.5 Hz), with its frequency doubling, approaches BPFO (2~9BPFO). Then, it can be inferred that the outer race fault has occurred.

(B) MOMEDA-TKEO method

**Figure 10:**
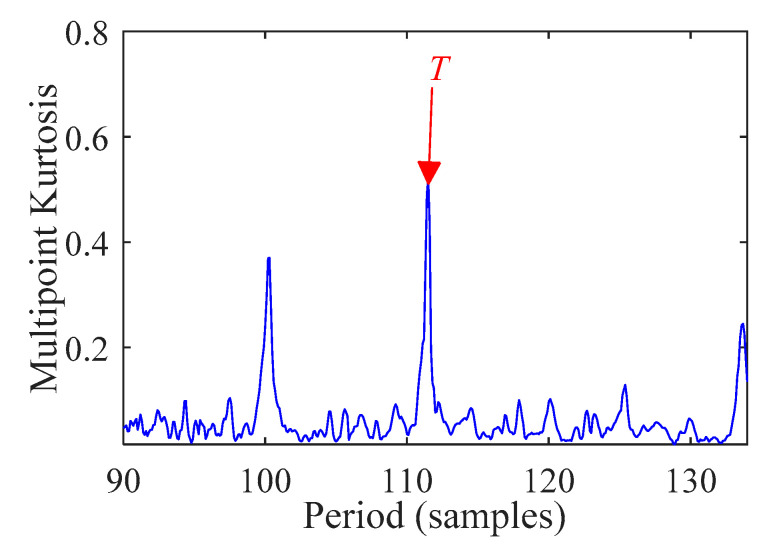
Multipoint kurtosis spectrum.

**Figure 11:**
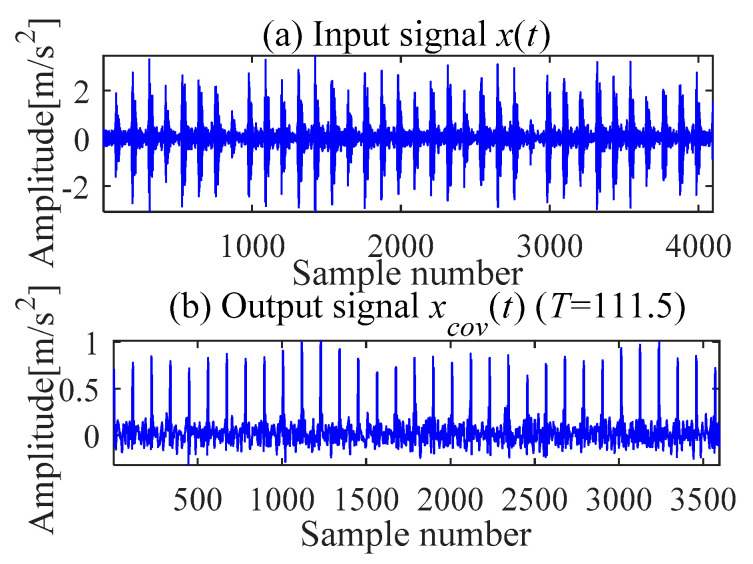
Signal deconvolution (MOMEDA).

According to [38] and [30], we analyze the outer race fault signal with the fault period *T* = (90,134) and filter length *L* = 500. The multi-point kurtosis diagram is shown in Figure 10. When the multi-point kurtosis reaches the maximum value, the fault period *T* is 111.5. The optimal deconvolution signal *x_cov_*(*t*) is presented in Figure 11.

**Figure 12:**
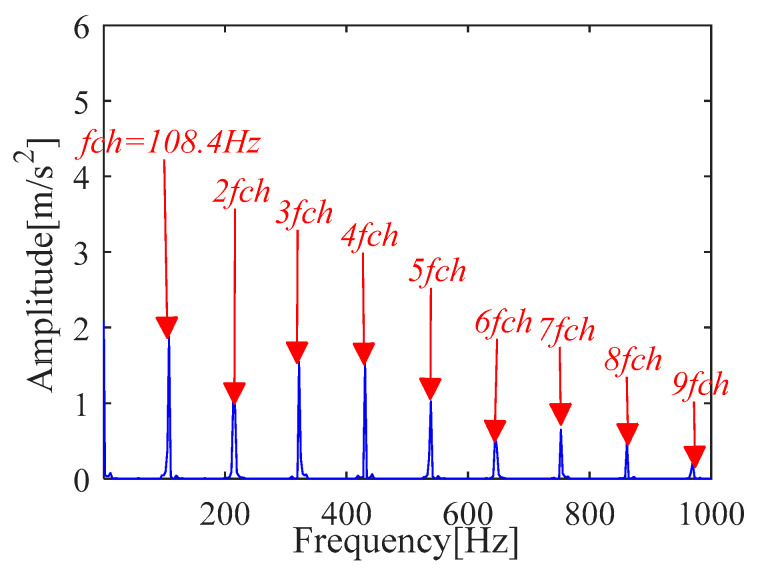
Teager-Kaiser energy spectrum (*x*_*_cov_*(*t*)).

The *x_cov_*(*t*) is demodulated by the TKEO and the corresponding spectrum is shown in Figure 12 where the result shows the characteristic frequency (108.4 Hz), with its frequency doubling, approaches BPFO (2~9BPFO). Further, it is inferred from the original signal that there exists an outer ring fault.

(C) FK method

**Figure 13:**
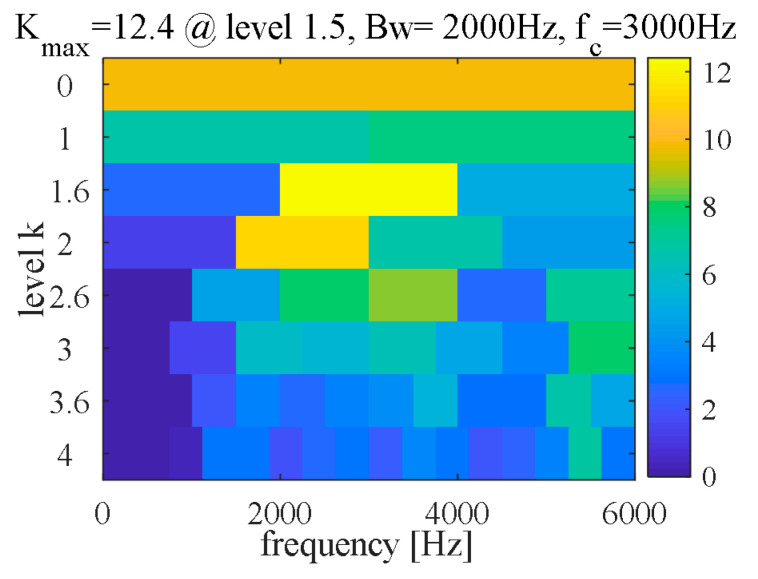
Fast kurtogram (outer race).

**Figure 14:**
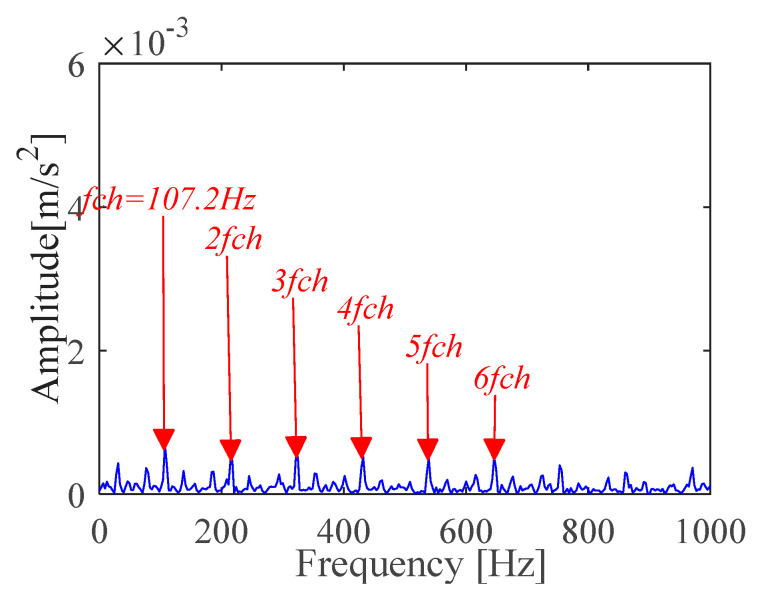
Fault characteristic spectrum (FK).

Fast kurtogram (FK) [48] is a time-frequency analysis method for spectral kurtosis that shows sufficient efficiency in transient fault detection and has been widely used in fault diagnosis field. Therefore, FK is used as a reference for comparative analysis in this paper. The FK and fault characteristic spectrum are shown in Figure 13 and Figure 14, respectively. The result shows the characteristic frequency (107.2 Hz) with its frequency doubling (2~6BPFO) can be extracted.

**Figure 15:**
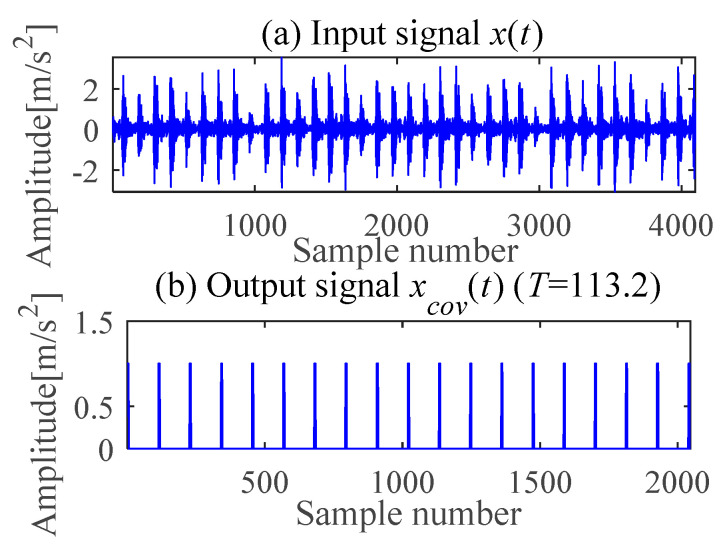
Signal deconvolution (L = 2050).

**Figure 16:**
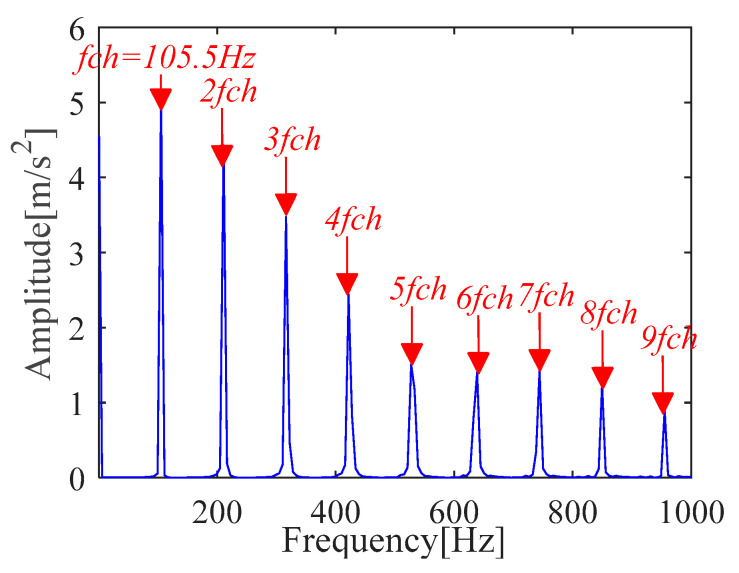
Teager-Kaiser energy spectrum (L = 2050).

Compared with the MOMEDA-TKEO method and FK method, the amplitude obtained by the IMOMEDA-TKEO is clearer. Test 3 is carried out through the filter length optimized by the proposed method, without changing the fault period *T* search interval of traditional MOMEDA. Then, *x_cov_*(*t*) and its Teager-Kaiser energy spectrum are shown in Figure 15 and Figure 16, respectively.

**Figure 17:**
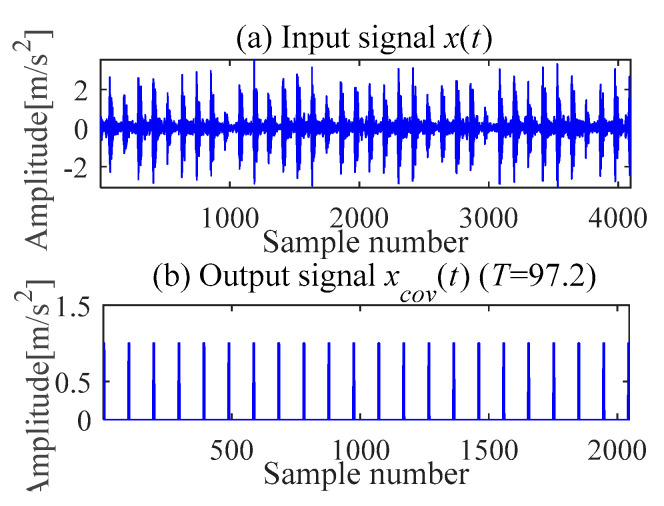
Signal deconvolution (pseudo period T = 97.2).

**Figure 18:**
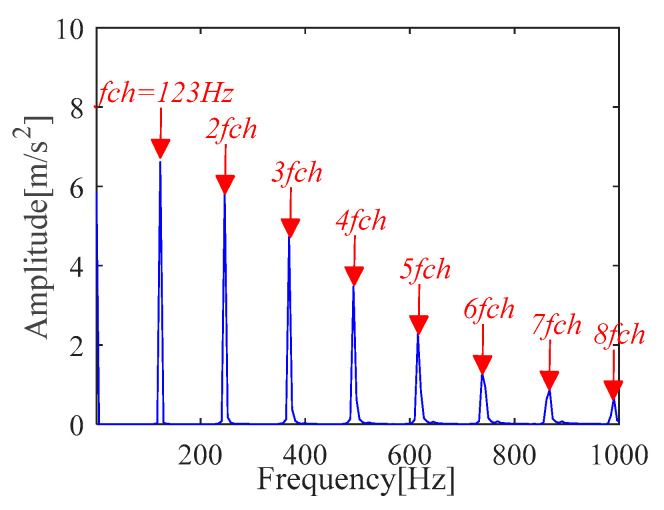
Teager-Kaiser energy spectrum (T = 97.2).

It can be seen from the Mkurt spectrum that the search interval of fault period *T* directly affects the denoising effect of MOMEDA. However, the interval of *T* depends on prior knowledge and has trial and error. When the original signal is affected by complex background noise, the Mkurt spectrum may not provide correct fault period information according to the location of maximum multipoint kurtosis. For example, we get the pseudo fault period as *T =* 97.2. Furthermore, the optimal periodic pulse signal *x_cov_*(*t*) cannot be obtained, as shown in Figure 17. The deconvolution effect is not ideal. The wrong fault characteristic frequency is 123 Hz and its spectrum is shown in Figure 18.

In order to further confirm the effectiveness of the proposed method quantitatively, a new signal-to-noise ratio (SNR) [49] is used as an index for Teager-Kaiser energy spectrum to complete the quantitative comparative analysis among the IMOMEDA-TKEO, MOMEDA-TKEO and the FK. The new SNR is defined as follows.

(23) $${SNR}_{fd}{=10\log}_{10}\frac{\sum_{i=1}^{M}p(i\cdot f_{d})}{\sum_{f=0}^{M\cdot f_{d}}p(f)-\sum_{i=1}^{M}p(i\cdot f_{d})}$$

where *f*_d_ is the fault characteristic frequency, *M* is the multiple of the fault characteristic frequency; *p*(*f*) is the Teager-Kaiser energy spectrum [50]. It can be seen from Table 3 that the *SNR_fd_* index of the presented method is the highest, which indicates that the proposed method has better ability in weak feature representation.

#### 4.1.2. Inner Race Fault Feature Extraction

**Figure 19:**
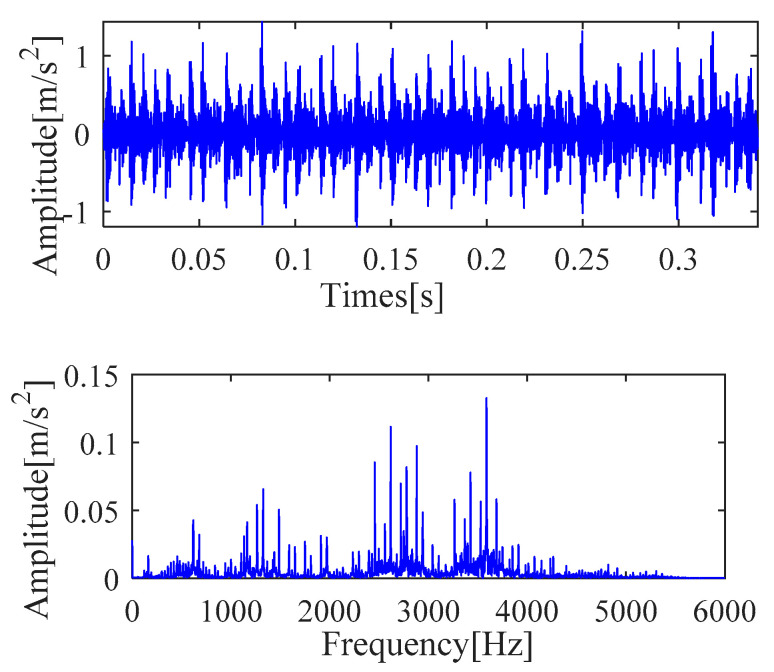
Vibration signal time and frequency analysis.

Figure 19 shows the time-domain and frequency-domain analysis results of the original inner race signal, respectively. It is seen from Figure 19 that these waveforms cannot directly obtain the information related to the inner race fault. Further analysis is required to determine the fault characteristic frequency and other details. Therefore, IMOMEDA-TKEO and MOMEDA-TKEO are proposed to complete subsequent analysis.

**Figure 20:**
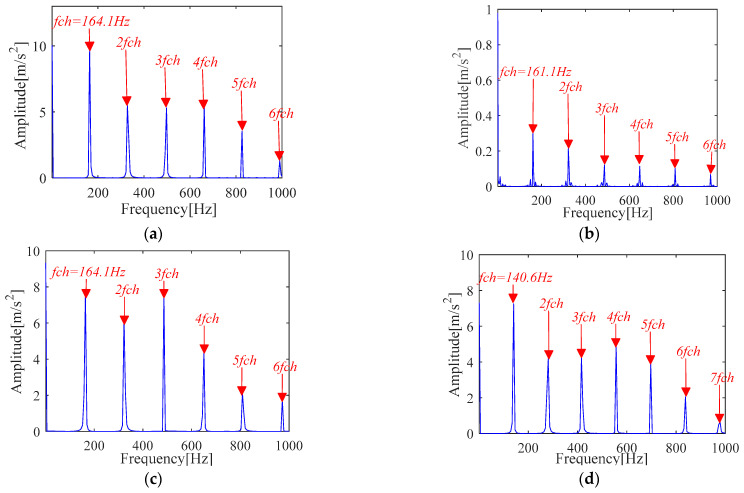
Teager-Kaiser energy spectrum (inner race) (**a**) IMOMEDA-TKEO, *T* = 75, *L* = 2100 (**b**) MOMEDA-TKEO, *T* = (59,88), *L* = 500 (**c**) MOMEDA-TKEO, *T* = (59,88), *L* = 2100 (**d**) MOMEDA-TKEO, pseudo fault period *T* = 86, *L* = 2100.

**Figure 21:**
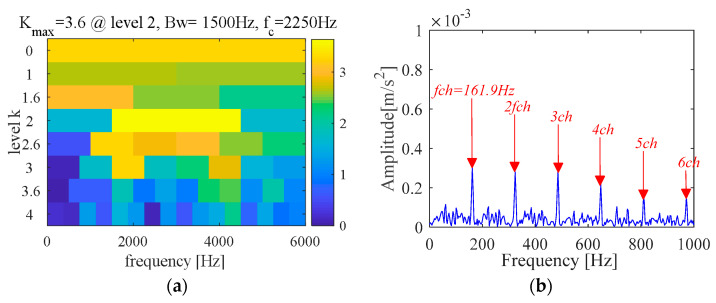
Vibration signal analysis results based on FK (**a**) Fast kurtogram (inner race) (**b**) Fault characteristic spectrum (FK).

The specific experimental process of IMOMEDA-TKEO, MOMEDA-TKEO and the FK method is the same as Section 4.1.1. The experimental comparison results are shown in Figure 20 and Figure 21.

The proposed method can achieve comparable effects as the MOMEDA-TKEO method as shown in Figure 20a,b. They both can extract the inner race fault features from the original vibration signals. It further proves the effectiveness of two methods.

Under the condition that the fault period *T* is invariant, filter length *L* is obtained by the proposed method and presented in Figure 20c. It can be seen from Figure 20c that the amplitude of TKEO spectrum apparently increases, which indicates filter length *L* directly affects the deconvolution performance of the MOMEDA method. In addition, it can be seen from Figure 20d that the selection of fault period *T* directly affects the performance of the method.

FK analysis results are shown in Figure 21. Compared with Figure 20, similar conclusions can be obtained, but the fault characteristic frequency with its frequency doubling characteristic obtained by IMOMEDA-TEO method are more prominent and clearer than those obtained by FK method.

From Table 4, the *SNR_fd_* for the IMOMEDA method is higher, which further implies the IMOMEDA has the qualitative and quantitative advantage over the original MOMEDA algorithm and FK method.

### 4.2. Case 2: NASA Data Analysis

In order to verify the good generalization ability of the IMOMEDA-TKEO method, the further experiments were carried out with NASA bearing datasets.

**Figure 22:**
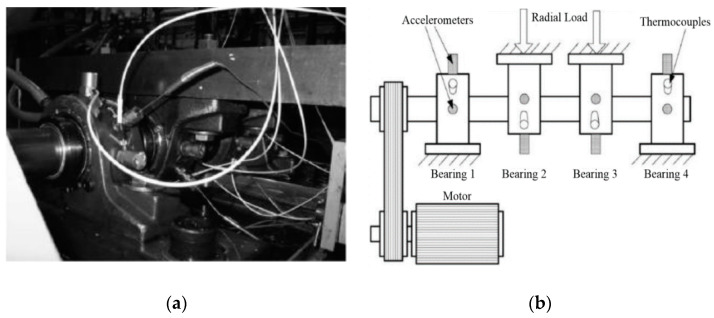
Bearing test rig of NASA data center (**a**) Test platform (**b**) Sensor layout.

The test-bed shown in Figure 22 is composed of four bearings, rotating shaft, two acceleration sensors and electronic control unit. The test bearings are located in the motor shaft. Some accelerometers sensors are placed to the motor housing. The motor speed is 2000 rpm and the digital data is collected by sampling with the frequency of 20 kHz. The detailed measured parameters of the bearing are shown in Table 5.

From the bearing parameters shown in Table 5, according to Equation (19) and Equation (20), the fault characteristic frequency BPFO = 236.4 Hz for outer race and BPFI = 296.93 Hz for inner race can be calculated, respectively, and the data length *N* is 4096 points.

**Figure 23:**
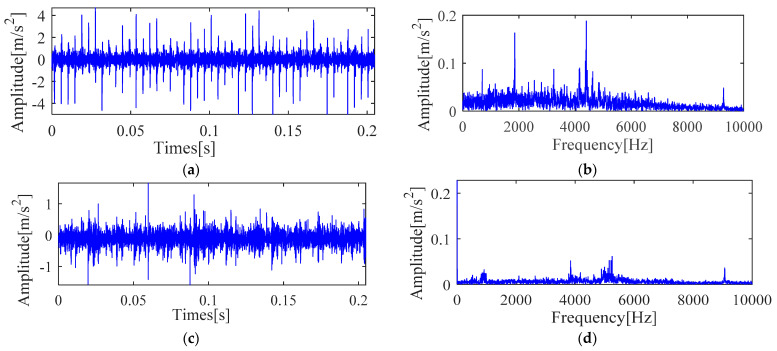
Vibration signal time and frequency analysis (**a**) Time domain waveform (outer race) (**b**) Frequency domain waveform (outer race) (**c**) Time domain waveform (inner race) (**d**) Frequency domain waveform (inner race).

**Figure 24:**
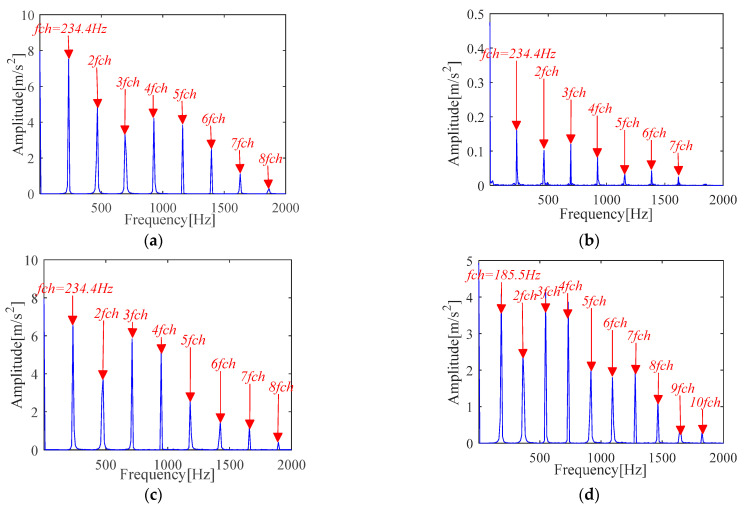
Teager-Kaiser energy spectrum (outer race) (**a**) IMOMEDA-TKEO, *T* = 86, *L* = 2100 (**b**) MOMEDA-TKEO, *T* = (68,102), *L* = 500. (**c**) MOMEDA-TKEO, *T* = (68,102), *L* = 2100 (**d**) MOMEDA-TKEO, pseudo fault period *T* = 99, *L* = 2100.

**Figure 25:**
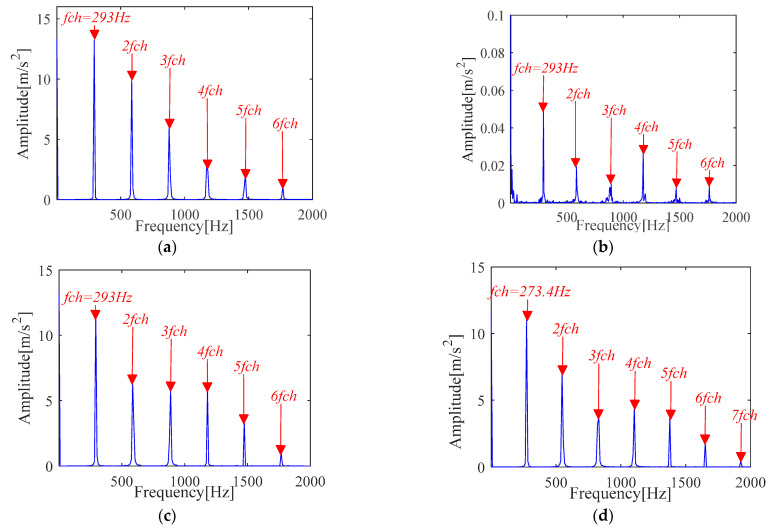
Teager-Kaiser energy spectrum (inner race) (**a**) IMOMEDA-TKEO, *T* = 69, *L* = 2050 (**b**) MOMEDA-TKEO, *T* = (54,81), *L* = 500 (**c**) MOMEDA-TKEO, *T* = (54,81), *L* = 2050 (**d**) MOMEDA-TKEO, pseudo fault period *T* = 73, *L* = 2050.

Experimental comparative analysis is complete by utilizing the vibration signals of outer race and inner race. Furthermore, the vibration signal time and frequency analysis is presented in Figure 23. It is seen from Figure 23 that these waveforms cannot directly determine the fault characteristic frequency and other details. In order to further analyze the fault information of the bearing, the same implementation process as Section 4.1 is taken to achieve the deconvolution results of the outer race and inner race, respectively, as shown in Figure 24 and Figure 25.

The experimental results of Figure 24 and Figure 25 show that the proposed IMOMEDA-TKEO method effectively extracts fault features of outer race and inner race and achieves significantly better processing performance than the MOMEDA-TKEO method.

**Figure 26:**
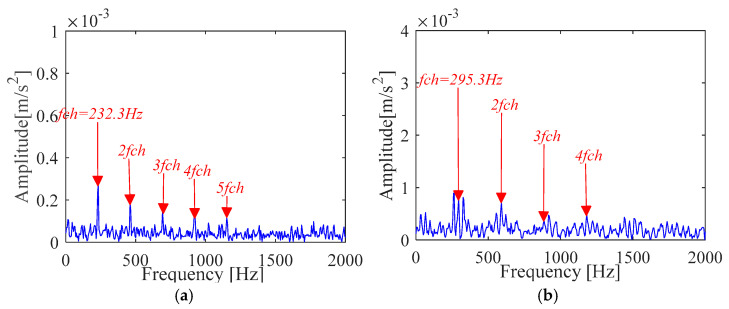
Vibration signal analysis results based on FK (**a**) Fault characteristic spectrum(outer race) (**b**) Fault characteristic spectrum(inner race).

FK based method can extract fault features of outer race and inner race from the original vibration signals. Unfortunately, it can be seen from Figure 26 that the obtained fault characteristic frequency is not obvious (the amplitude is far less than the other two methods) and contains more noise components. Compared with Figure 24 and Figure 25, the filtering performance is poorer than the IMOMEDA-TKEO method.

In order to intuitively compare the effectiveness of the method, the *SNR_fd_* index is calculated and shown in Table 6. By comparing and analyzing the data in the Table 6, the conclusions similar to Section 4.1 can be drawn.

### 4.3. Application Testing

**Figure 27:**
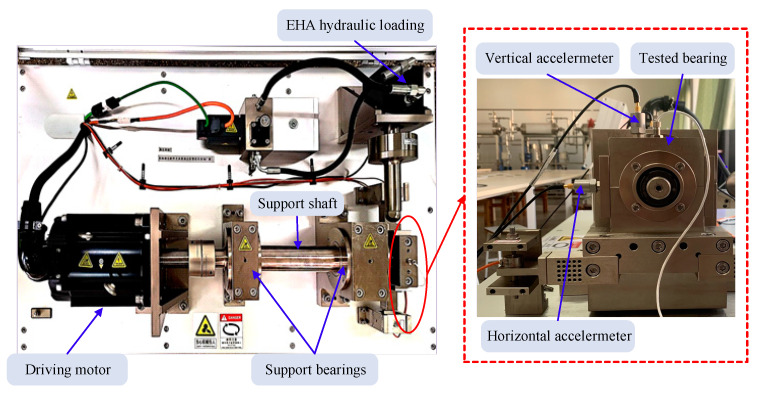
Bearing testbed.

In order to further prove the validity of the proposed method, the practical test data from the self-made KUST-SY experiment platform is selected for subsequent analysis. The platform is able to simulate different failure conditions of rolling bearings. The structure of the test rig is shown in Figure 27, which consists of driving motor, rotating shaft, support bearing, hydraulic loading system, accelerometers and tested bearing. The device can realize radial hydraulic loading and the rotation speed is controlled by computer. Accelerometers are installed in the vertical and horizontal directions of the test bearing base to collect the bearing vibration signal. Because the load is applied in the horizontal direction, the accelerometer placed in this direction is capable of capturing more fault information of the tested bearings. Therefore, the horizontal vibration signals are selected to analyze bearing fault characteristics.

**Figure 28:**
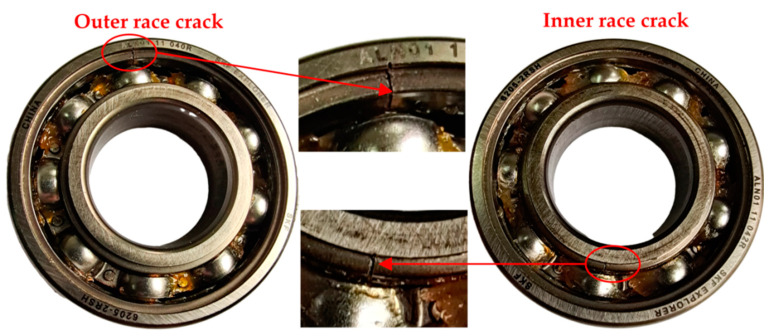
Fault bearings.

The failure behaviors of bearing system are diverse, including stripping, wear, fracture, crack, electrical erosion, indentation for the inner race and outer race. As displayed in Figure 28, in this experiment, inner ring crack and out ring crack of the bearing have been mainly simulated considering the existing experimental conditions. The raceway surface cracks (the fault diameter is 0.2 mm) of the inner ring and outer ring have an artificial linear cutting with fiber laser cutter. The sampling frequency is 51.2 kHz and the motor speed is 1797 rpm. The sampling interval is 20 s and the sampling time is 10 s. The tested bearing model is 6205-2RSH, and its relevant parameters are shown in Table 7. According to Table 7, the fault characteristic frequency BPFO = 107.22 Hz for the outer race and BPFI = 162.33 Hz for the inner race can be calculated, respectively.

**Figure 29:**
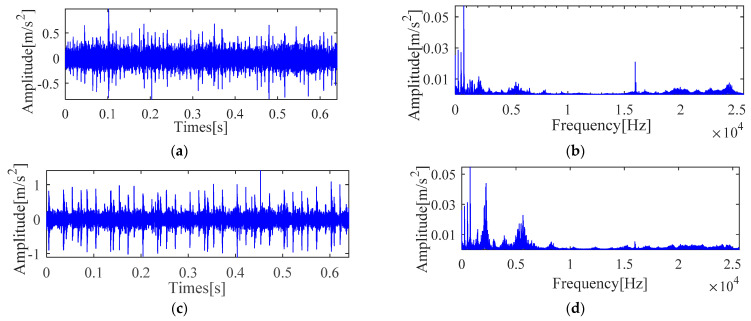
Vibration signal time and frequency analysis (**a**) Time domain waveform (outer race) (**b**) Frequency domain waveform (outer race) (**c**) Time domain waveform (inner race) (**d**) Frequency domain waveform (inner race).

**Figure 30:**
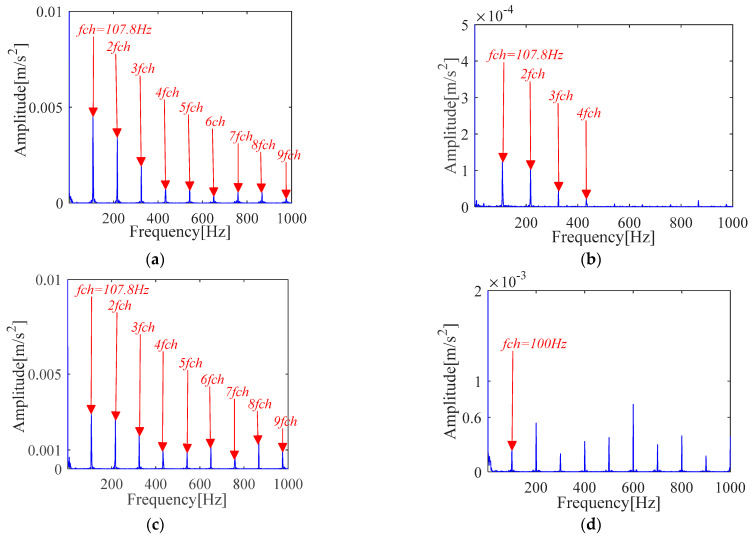
Teager-Kaiser energy spectrum (outer race) (**a**) IMOMEDA-TKEO, *T* = 472, *L* = 2200 (**b**) MOMEDA-TKEO, *T* = (382,573), *L* = 500 (**c**) MOMEDA-TKEO, *T* = (382,573), *L* = 2200 (**d**) MOMEDA-TKEO, pseudo fault period *T* = 512, *L* = 2200.

**Figure 31:**
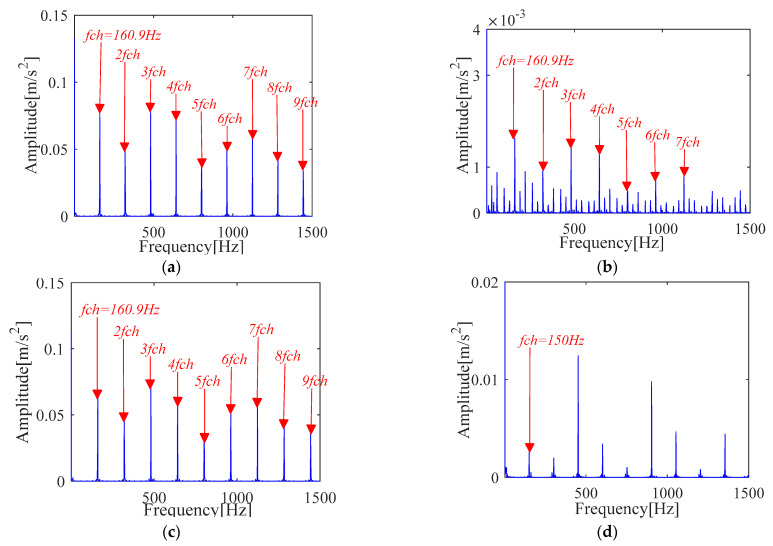
Teager-Kaiser energy spectrum (inner race) (**a**) IMOMEDA-TKEO, *T* = 319, *L* = 2400 (**b**) MOMEDA-TKEO, *T* = (252,378), *L* = 500 (**c**) MOMEDA-TKEO, *T* = (252,378), *L* = 2400 (**d**) MOMEDA-TKEO, pseudo fault period *T* = 340, *L* = 2400.

**Figure 32:**
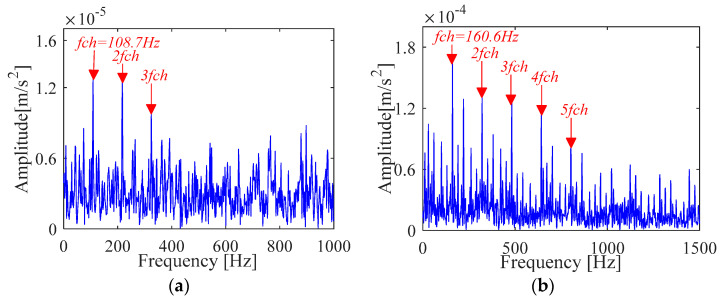
Vibration signal analysis results based on FK(**a**) Fault characteristic spectrum (outer race) (**b**) Fault characteristic spectrum (inner race).

The time and frequency analysis is shown in Figure 29 for the vibration signal of the inner ring and outer ring (The length of data-set *N* is 32768). We cannot get valuable information related to fault characteristic frequency from Figure 29, which is the same as previous cases. Therefore, the fault information is determined by the same implementation process with Section 4.1 and plotted in Figure 30, Figure 31 and Figure 32, respectively.

It can be seen from Figure 30 and Figure 31 that the fault features extracted by the proposed IMOMEDA-TKEO method are more obvious in the Teager energy spectrum. At the same time, it can be seen from Figure 30d and Figure 31d that the improper period *T* will lead to erroneous results in the MOMEDA method.

The fault characteristic frequency for the inner race and outer race extracted by FK is shown in Figure 32. Comparing with the fault characteristic spectrum extracted by MOMEDA based method as shown in Figure 30 and Figure 31, it can be seen that the fault characteristic frequencies can be accurately obtained by FK. However, there are many interference components in the spectrum, which result in the faint characteristic amplitude. In particular, the outer race seems to be more seriously disturbed by noise, and only three times of double frequency is extracted, while the other two methods can extract nine times of double frequency. In addition, through the comparison of performance indicator *SNR_fd_* presented in Table 8, it is also found that the IMOMEDA-TKEO method can achieve better performance than the MOMEDA-TKEO method and FK method in noise reduction.

### 4.4. Comparative Analysis with Three Cases

Through the comparative experimental analysis of IMOMEDA-TKEO, MOMEDA-TKEO and FK, the following can be summarized:

(a) Combining with three case studies, the experimental results show that the IMOMEDA-TKEO method is better than the other method described in Table 1, which enhances the application prospect of the proposed method.

(b) In this experiment, we select execution time and iterations to evaluate the complexity of the optimal parameter selection method and all the experiments are programmed in MATLAB 2018a on an Intel(R) Core(TM) i5-7300HQ CPU @ 2.50 GHz Laptop with 16.00 GB RAM. The parameter optimization time and iterations of IMOMEDA algorithm and the main program execution time of the comparative methods are shown in Table 9 and Table 10, respectively. It is seen from Table 9 that we can calculate fault period *T* of MOMEDA quickly with little time. Compared with the traditional MOMEDA method in which the parameters are set to the default value, the grid search method based on *EHNR* spectral entropy for selecting optimal filter length *L* does consume some time. However, optimization algorithm itself is not the main reason of time-consuming. The main reason is that as the iterations increases, the MOMEDA filter time keeps accumulating, leading to more training time of filter length. The purpose of this paper is to accurately calculate the fault period *T* of MOMEDA and select the optimal filtering length *L* of MOMEDA. The rationality and necessity of MOMEDA parameter selection are demonstrated on three different datasets. Although the calculation needs a certain time, it is within the acceptable range.

(c) It can be seen from Table 10 that the FK method and the traditional MOMEDA method (default filter length and fault period) take less time. However, when dealing with abnormal or unknown vibration signals, the default parameters of MOMEDA are obviously not feasible. It is verified that the time-consuming by the trial and error to determine the MOMEDA parameters is almost the same as that of the proposed method. However, the result of unreasonable parameter setting is much worse than IMOMEDA, such as Figure 24d and Figure 25d, which also proves the significance of optimizing MOMEDA parameters. The proposed IMOMEDA-TKEO method can effectively estimate the period *T* of vibration signal, and set the filter length *L* reasonably by using the improved grid search method. The execution time is within the acceptable range.

## 5. Discussion

(a) For the traditional MOMEDA method, the range of *T* has to be set by a trial and error method. Furthermore, the range of *T* selected in the above experiments is the best result obtained by multiple attempts. If the selected range changes, the pseudo period will cause misdiagnosis. The proposed method introduces the *EHNR* spectrum to calculate the signal period *T* quickly and optimizes the filter length *L* by the improved grid search method. It transforms the iterative optimization problem into a single dimension optimization problem. Hence, the proposed method increases the operation efficiency and improves the deconvolution performance of MOMEDA method.

(b) In the proposed method, the search range of the filter length *L* is set as [34]. Only the lower limit of the filter length *L* is given, while there is no upper limit, which makes the search range larger and the algorithm run for a long time. According to the specific characteristics of the signal, optimizing the search range can improve the efficiency of the algorithm. In addition, if we can obtain the corresponding relationship and change rule between the MOMEDA filter length *L* and period *T* on the basis of (a), it will further improve the operation efficiency and the practical application value of the proposed method.

(c) Compared with other types of signal characteristics, the practical test vibration signals for inner race from the rolling bearing fault simulation experiment platform are not only disturbed by noise, but also by harmonic signal of the outer race and rolling element. Under strong background noise, the IMOMEDA-TKEO method has been demonstrated to significantly outperform traditional signal processing methods in terms of noise reduction performance and fault feature representation robustness, which boosts the generalization ability.

## 6. Conclusions

In this paper, a rolling bearing fault feature extraction method based on the IMOMEDA-TKEO is proposed. Some conclusions are obtained as follows:

(1) The *EHNR* method is introduced to obtain the *EHNR* spectrum of the original signal and solves the problem that the fault period *T* of MOMEDA depends on prior knowledge.

(2) The *EHNR* spectral entropy is proposed as the objective function, and the improved grid search method is introduced to solve the filter length *L* selection in MOMEDA.

(3) Comparative experimental analysis of the proposed method with the MOMEDA-TKEO method and the FK method are completed. The proposed method can achieve the best filtering performance, which verifies the effectiveness and feasibility of the IMOMEDA-TKEO.

In the proposed method, the upper limit of the filter length *L* is not given, which makes the execution time and iterations of the algorithm take a certain time (it is within the acceptable range). In future studies, we will focus on improving the calculation accuracy of fault period *T* for complex signals and optimizing the search range of filter length *L*. We will also explore the change rule between the filter length *L* and fault period *T* to improve the performance of MOMEDA.

## Figures and Tables

**Table 1:** The brief overview of the comparative experiment.

Test	Algorithm	Parameters Optimization	Data Sets
Test 1	IMOMEDA-TKEO	The *EHNR* spectrum is utilized to calculate fault period *T* and the improved grid search method base on *EHNR* spectral entropy is proposed to determine optimal filter length *L*.	(a) CWRU data sets (b) NASA data sets (c) KUST-SY data sets
Test 2	MOMEDA-TKEO-class 1	According to [38], set the default range of *T* is (0.8*T_α_*,1.2*T_α_*), *T_α_* = *f*_s_/*f*_cα_,Where *f*_s_ is sampling frequency and *f*_cα_ is fault frequency. The default filter length *L* [30] is put to 500.	
Test 3	MOMEDA-TKEO-class 2	The value range of *T* is consistent with Test 2 and the method to determine *L* follows Test 1.	
Test 4	Fast kurtogram	Spectral kurtosis is taken as the function of Short Time Fourier Transform (STFT) window width to search the optimal filter parameters.	

**Table 2:** Bearing basic parameters.

**Rolling Bearing-6205-2RSJEMSKF**	**Rolling Element Number (** ***Z*** **)**	**Inner Diameter** **(Inches)**	**Outer Diameter** **(Inches)**	**Rolling Element Diameter ** ***d*** ** (Inches)**	**Limiting Speed (rpm)**
	9	0.9843	2.0472	0.3126	18,000
	**Contact angle (** ***θ*** **)**	**Pitch circle diameter ** ***D*** ** (inches)**	**Dynamic load rating (** ***N*** **)**	**Static load rating (** ***N*** **)**	**/**
	0°	1.537	14,800	7800	/

**Table 3:** The value of *SNR_fd_* with different methods.

Method	IMOMEDA-TKEO (*T =* 112; *L =* 2050)	MOMEDA-TKEO-Class 1 (*T =* (90,134); *L =* 500)	MOMEDA-TKEO-Class 2 (*T =* (90, 134); *L =* 2050)	Fast Kurtogram
Value of *SNR_fd_*	2.6600	0.2352	2.5014	0.1784

**Table 4:** The value of *SNRfd* with different methods.

Method	IMOMEDA-TKEO (*T* = 75; *L* = 2100)	MOMEDA-TKEO-Class 1 (*T* = (59,88); *L* = 500)	MOMEDA-TKEO-Class 2 (*T* = (59, 88); *L* = 2100)	Fast Kurtogram
Value of *SNR_fd_*	1.4538	0.7664	1.4200	0.3632

**Table 5:** Bearing foundation parameters.

Rolling Element Number (*Z*)	Contact Angle (*θ*)	Rolling Element Diameter *d* (mm)	Pitch Diameter *D* (mm)	Rotational Speed (rpm)	Dynamic Load Rating (*N*)	Static Load Rating (*N*)	Limiting Speed (rpm)
16	15.17	0.331	2.815	2000	6500	7470	2500

**Table 6:** The value of *SNR_fd_* with different methods.

**Outer race**	**Method**	**IMOMEDA-TKEO** (***T***** = 86; *****L***** = 2100)**	**MOMEDA-TKEO-class 1** (***T***** = (68,102); *****L***** = 500)**	**MOMEDA-TKEO-class 2 (** ***T*** ** = (68,102); ** ***L*** ** = 2100)**	**Fast kurtogram**
	Value of *SNR_fd_*	1.9344	0.1675	1.8167	0.1139
**Inner race**	**Method**	**IMOMEDA-TKEO** (***T***** = 69;*****L***** = 2050)**	**MOMEDA-TKEO-class 1** (***T***** = (54,81); *****L***** = 500)**	**MOMEDA-TKEO-class 2 (** ***T*** ** = (54,81); ** ***L*** ** = 2050)**	**Fast kurtogram**
	Value of *SNR_fd_*	1.4221	0.1127	1.2527	0.1105

**Table 7:** Bearing foundation parameters.

Rolling Element Number (*Z*)	Contact Angle (*θ*)	Bearing Pitch Diameter (mm)	Roller Diameter (mm)	Dynamic Load Rating (*N*)	Static Load Rating (*N*)	Limiting Speed (rpm)
9	0	39	8	14,800	7800	18,000

**Table 8:** The value of *SNR_fd_* with different methods.

**Outer Race**	**Method**	**IMOMEDA-TKEO** (***T***** = 472; *****L***** = 2200)**	**MOMEDA-TKEO-class 1 (** ***T*** ** = (382,573); ** ***L*** ** = 500)**	**MOMEDA-TKEO-class 2 (** ***T*** ** = (382,573); ** ***L*** ** = 2200)**	**Fast kurtogram**
	Value of *SNR_fd_*	2.8843	0.5228	1.1936	0.2830
**Inner Race**	**Method**	**IMOMEDA-TKEO** (***T***** = 319; *****L***** = 2400)**	**MOMEDA-TKEO-class 1 (** ***T*** ** = (252,378); ** ***L*** ** = 500)**	**MOMEDA-TKEO-class 2 (** ***T*** ** = (252,378); ** ***L*** ** = 2400)**	**Fast kurtogram**
	Value of *SNR_fd_*	2.9423	0.5179	1.6656	0.2961

**Table 9:** The parameter optimization time and iterations of IMOMEDA algorithm.

Fault Type	Algorithm	Fault Period *T*/Time	Fault Period *T*/Iterations	Filter Length *L*/Time	Filter Length *L*/Iterations
CWRU(outer)	IMOMEDA	0.465s	8	57.114s	76
CWRU(inner)		0.751s	9	57.950s	77
NASA(outer)		0.594s	9	64.015s	76
NASA(inner)		0.690s	9	57.617s	77
KUST-SY(outer)		0.744s	6	81.343s	61
KUST-SY(inner)		0.756s	6	92.087s	67

**Table 10:** Main program execution time of the comparative methods.

	Datasets	CWRU (Outer)	CWRU (Inner)	NASA (Outer)	NASA (Inner)	KUST-SY (Outer)	KUST-SY (Inner)
Algorithm							
IMOMEDA		65.385s	63.792s	68.859s	61.770s	83.616s	102.741s
		(*T* = 112)	(*T* = 75)	(*T* = 86)	(*T* = 68)	(*T* = 472)	(*T* = 319)
		(*L* = 2050)	(*L* = 2100)	(*L* = 2100)	(*L* = 2050)	(*L* = 2200)	(*L* = 2400)
MOMEDA-TKEO-class 1		1.127s	1.334s	1.181s	1.171s	10.179s	7.389s
		(*T* = (90,134))	(*T* = (59,88))	(*T* = (68,102))	(*T* = (54,81))	(*T* = (382,573))	(*T* = (252,378))
		(*L* = 500)	(*L* = 500)	(*L* = 500)	(*L* = 500)	(*L* = 500)	(*L* = 500)
MOMEDA-TKEO-class 2		4.221s	4.357s	4.685s	4.562s	21.490s	22.958s
		(*T* = (90,134))	(*T* = (59,88))	(*T* = (68,102))	(*T* = (54,81))	(*T* = (382,573))	(*T* = (252,378))
		(*L* = 2050)	(*L* = 2100)	(*L* = 2100)	(*L* = 2050)	(*L* = 2400)	(*L* = 2000)
FK		0.809s	0.763s	0.825s	0.837s	9.543s	8.906s

## Data Availability

Data is available on Reference [46] and Reference [47].

## Abbreviations

MOMEDA-multipoint optimal minimum entropy deconvolution adjusted	TFMSR-time-delayed feedback monostable stochastic resonance
IMOMEDA-improved MOMEDA	LSTM-long and short time memory
*EHNR*-envelope harmonic-to-noise ratio	PSO-particle swarm optimization
GOA-grasshopper optimization algorithm	CVS-continuous vibration separation
MED-minimum entropy deconvolution	ESK-envelope spectrum kurtosis
FK-fast kurtogram	STFT-Short Time Fourier Transform
MCKD-maximum correlation kurtosis deconvolution	NASA-National Aeronautics and Space Administration
AC-autocorrelation function	CWRU-Case Western Reserve University
TKEO-Teager-Kaiser energy operator	BPFO-Outer ring defect frequency
SNR-signal-to-noise ratio	BPFI-Inner ring defect frequency
